# Sulfo-phospho-vanillin method for screening *Aurantiochytrium* strains with high docosahexaenoic acid levels

**DOI:** 10.1186/s13568-025-01859-9

**Published:** 2025-03-20

**Authors:** Person Pesona Renta, Ta-Yu Huang, Ping-Hao Yu, Anna C.-C. Jang, Yi-Min Chen

**Affiliations:** Department of Biotechnology and Bioindustry Sciences, National Cheng Kung University, Tainan, Taiwan

**Keywords:** *Aurantiochytrium*, Docosahexaenoic acid (DHA), High-throughput screening, Sulfo-phospho-vanillin (SPV) method, UV mutagenesis

## Abstract

*Aurantiochytrium* are protists found in marine and estuarine environments, known for producing high quantities of omega-3 fatty acids, particularly docosahexaenoic acid (DHA). The commercial viability of this species is currently hindered by the lack of reliable screening methods for the rapid identification of strains with high DHA content. This study developed a high-throughput screening platform based on the sulfo-phospho-vanillin (SPV) reaction, which produces a pink chromophore upon reacting with C–C double bonds in lipids. Analysis of 200 strains derived through the UV mutagenesis of the *Aurantiochytrium limacinum* strain BL10 revealed 7 strains that exhibited significantly elevated SPV reactivity, compared to the naïve strain (*P* < 0.01). Gas chromatography-mass spectrometry analysis revealed that in 4 of the 7 strains, DHA levels were significantly higher than those of the naïve strain. Among those 4 strains, the SPV reactivity values of two strains were precisely correlated with their DHA/DPA content. The SPV reaction protocol proposed in this paper has considerable potential for the high-throughput screening of *Aurantiochytrium* strains particularly those derived through UV mutagenesis.

**Graphical abstract:**

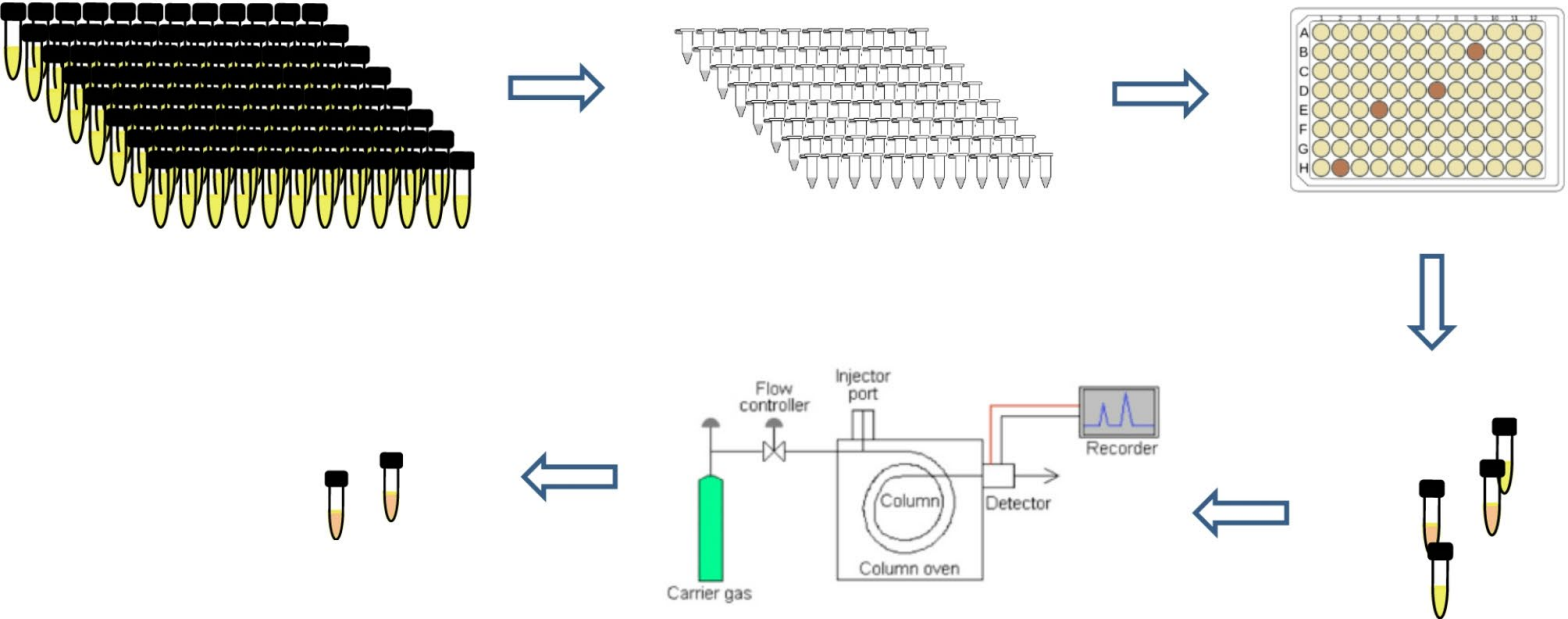

**Supplementary Information:**

The online version contains supplementary material available at 10.1186/s13568-025-01859-9.

## Introduction

Docosahexaenoic acid (DHA) is a long-chain omega-3 highly unsaturated fatty acid (HUFA) characterized by 22 carbon atoms and six cis double bonds, with the first double bond positioned at the third carbon from the omega end of the fatty acid chain (Clauss and Rankin 2021). DHA plays a crucial role in the structure and function of cellular membranes, particularly in the brain (Lauritzen et al. 2016; Petermann et al. 2022) and retina (Sugasini et al. 2020; Swinkels and Baes 2023). DHA has been implicated in cognitive development, visual acuity, and overall neurological health (Rodrigues et al. 2023; Uauy et al. 2001; Weiser et al. 2016). DHA has also been linked to anti-inflammatory processes as well as a reduced risk of cardiovascular disease (Heras-Sandoval et al. 2016). DHA is widely used in dietary supplements, infant formulas, and functional foods (Li et al. 2021; Swanson et al. 2012). Natural sources of DHA include fatty fish (e.g., salmon and tuna) and marine protists (e.g., microalgae and thraustochytrids) (Santigosa et al. 2023). Note that marine protists are widely used in the commercial production of algal-based DHA supplements as a sustainable, vegetarian-friendly alternative to fish-derived sources (Craddock et al. 2017; Sprague et al. 2016).

Thraustochytrids, such as *Aurantiochytrium limacinum* (formerly *Schizochytrium limacinum* (Raghukumar 2008) are excellent candidates for the environmental sustainable production of DHA (Byreddy 2016; Kaliyamoorthy et al. 2023), and the commercialization of thraustochytrid-derived DHA is gaining momentum (Kaliyamoorthy et al. 2023). Nonetheless, numerous researchers have noted the challenges in the commercial production of DHA, including the high cost (Russell et al. 2022; Udayan et al. 2023), difficulties in downstream processing (Zhang et al. 2022), and the gradual decrease in DHA production of thraustochytrid strains (Pora and Zhou 2012).

The sustainability of the microbial DHA industry depends on the ability to identify high-yield strains of *Aurantiochytrium* in nature or through genetic or metabolic engineering (Ma et al. 2022). Researchers must contend with the problem of isolating strains with high DHA content from among an enormous number of new isolates and mutant lines. There is a pressing need for semi-quantitative methods for the high-throughput assessment of candidate thraustochytrid strains prior to the precise but labor-intensive task of chemical analysis (e.g., gas chromatography).

Lipophilic fluorescent stains, such as Nile Red and BODIPY, have been extensively used in the screening of oleaginous microorganisms with elevated oil content, such as yeast (Miranda et al. 2020), microalgae, and thraustochytrids (Rumin et al. 2015). Unfortunately, these stains cannot be used to differentiate between saturated and unsaturated fats, due to their limited specificity for HUFA (Moreira et al. 2020; Morschett et al. 2016; Rumin et al. 2015; Wang et al. 2018). Sulfo-phospho-vanillin (SPV) reagents are able to selectively identify C = C double bonds in lipids, which makes them suitable for the screening of microorganisms abundant in unsaturated fatty acid (Anschau et al. 2017; Patel et al. 2019a). SPV has been used to assess the total lipid content in select human samples (e.g., serum or meibum) and microorganisms; however, it has not previously been used for the screening of microorganisms with high unsaturated lipid content.

The objective in this study was to develop and validate an SPV-based high-throughput screening method specifically for the rapid identification of *Aurantiochytrium* strains with elevated DHA content. After establishing the optimal operational conditions for the SPV reaction, we analyzed 200 mutant lines derived through the UV mutagenesis of *Aurantiochytrium limacinum* strain BL10 to identify those with the highest SPV reactivity. GC-MS was then used to confirm that the DHA content of the selected mutant lines was indeed higher than that of the naïve strain.

## Materials and methods

### Preparation of BL10 culture

The *Aurantiochytrium limacinum* strain BL10 used in the current study was an axenic strain isolated from a mangrove forest near Taipei City on the west coast of Taiwan in 2007 (Yang et al. 2010). This strain has been deposited in the Bioresource Collection and Research Center (BCRC) in Taiwan, with the strain number BCRC 980009. After establishing a pure culture of the strain, samples were cryopreserved until use. This involved selecting a well-isolated discrete colony of BL10 growing on a M3 agar plate (Chou et al. 2022) to be upscaled in a 500-mL sterile flask with 100 mL H3 medium (Chaung et al. 2012). The culture was then cultivated under shaking at 150 rpm at 27 °C for 3 days. Aliquots (1 mL) of the culture were transferred to 2-mL cryovials, followed by a quick but gentle mixing with 1 mL of 50% glycerol aqueous solution. Sample were sealed in vials, which were stored at 4 °C for 60 min and then at − 20 °C for 30 min prior to cryopreservation at − 80 °C.

Activating BL10 cryocell stock involved rapid thawing in a 37 °C water bath. Fifty microliters of the stock was transferred into a 50-mL glass culture tube with 5 mL H3 medium and cultivated in a shaker (150 rpm) at 27 °C for 24 h. Following activation, aliquots (50 µL) of the seed culture were transferred to 50-mL test tubes with 5-mL of GYSS medium (Chou et al. 2022) and cultivated in a shaker (150 rpm) at 27 °C for 72 h.

### UV metagenesis

Activated BL10 cells were subjected to four consecutive 1/10 serial dilutions using 10 ppt diluted natural seawater. A 50 µL sample of the diluted culture containing roughly 200 viable cells was evenly spread on an M3 agar plate using ten 4 mm diameter glass beads (*n* = 3). UV treatment involved exposing cells to 254 nm UV light using a gel documentation system (Major Science DI-01). This involved placing the agar plate upside down directly on the light panel of the device, positioning the cell layer 1 cm from the panel surface (light intensity 250 mW/cm²). This ensured that the cells on the surface of the agar medium were directly exposed to UV light without interference from the agar medium.

To determine the optimal UV mutagenesis treatment time, the plates were exposed to UV radiation for 0, 1, 5, 10, 20, 30, 40, 50, 60, 70, 80, 90, or 100 s. The optimal treatment time was defined as the duration that resulted in a mortality rate of 60–80% (Pora and Zhou 2012). Following UV treatment, cells were cultivated on agar plates at 27 °C until colonies were visible to the naked eye. The colonies were then individually selected for cryopreservation in accordance with the methods outlined in the previous section.

### Optimizing the sample size for the SPV reaction

BL10 cells were cultured in GYSS medium (1 ppt diluted seawater supplemented with 0.9% sodium sulfate) or modified GYSS (10 ppt natural seawater). This resulted in two BL10 cultures with identical biomass but significant different DHA and n-6 docosapentaenoic acid (C22:5n-6; n-6 DPA) contents (Chaung et al. 2012). Five test tubes of each medium were prepared for cultivation.

After combining the five cultures with the same medium (total volume 25 mL), 2 mL aliquots were evenly dispensed into two 1.5 mL Eppendorf tubes. The samples underwent centrifugation at 5000 x g for 5 min to remove the culture medium, after which 1 mL of deionized water was added to resuspend the cells. Various aliquots of the resulting suspension (10, 15, 20, or 25 µL) were respectively dispensed into 1.5 mL Eppendorf tubes. Note that each volume was dispensed twice, resulting in 8 tubes.

SPV reactions were performed using phosphovanillin reagent in accordance with methods outlined in previous study (Anschau et al. 2017). Before initiating SPV reactions, some of sample were centrifuged at 5000 x g for 5 min to the remove water, while other samples underwent the SPV reaction directly without water removal. SPV reactions were performed in accordance with a 2-stage process described (Anschau et al. 2017).

The first stage involved carbonium ion formation. Aliquots of culture fluid (10, 15, 20, and 25 µL) were dispensed into 1.5-mL Eppendorf tubes in triplicate. To each tube was added 200 µL of concentrated sulfuric acid, followed by incubation in a dry bath at 100 °C for 5 min. The samples were vigorously vortexed for 20 s and incubated again at 100 °C for an additional 5 min. The tubes were then cooled to room temperature for 15 min.

The second stage involved color reaction. To each cooled sample was added 500 µL of phosphoric acid-vanillin reagent solution. The mixtures were incubated in a dry bath at 37 °C for 15 min, followed by 45-minute incubation at room temperature in the dark.

SPV reactivity was assessed by transferring of each reaction product (150 µL) to a 96-well plate and measuring absorbance at 530 nm using a microplate reader. The absorbance values served as reference points in calculating the relative SPV reactivity of the two BL10 cultures (MGYSS medium/ GYSS medium) under various reaction conditions (different sample volumes; with or without water removal prior to the SPV reaction).

Optimal reaction parameters were determined by identifying the experiment group with relative SPV reactivity values that most closely aligned with the results of fatty acid analysis. Fatty acid analysis involved the centrifugation of BL10 culture samples (23 mL), followed by a single rinse using deionized water, and lyophilization to remove intracellular moisture. Sample preparation and GC-MS analysis were conducted in accordance with the methods outlined in our previous study (Yang et al. 2010). After determining the DHA and n-6 DPA content (grams per liter of culture volume) of the two BL10 cultures, the total moles of C=C double bonds originating from DHA and n-6 DPA per liter of culture volume was calculated using the following formula:

$$\begin{aligned}\text{Total moles of C}={\rm C\,double bonds}&={\rm W}_{\rm DHA} ({\rm gram})/328.5\times6\\&\quad+ {\rm W}_{{\rm DPA}} ({\rm gram})/330.5\times5.\end{aligned}$$

A relative SPV reactivity value was derived from the relative number of moles of C = C double bonds in the two cultures. We expected that this *theoretical SPV reactivity value* would be positively correlated with the total moles of C=C double bonds in a given sample. This value was used as a reference value in comparing the relative SPV reactivity values obtained under the various reaction conditions.

### High-throughput screening of BL10 mutant lines based on SPV reactivity

All 200 mutant lines created via BL10 mutagenesis were activated and cultivated in accordance with the methods described in the section of preparation of BL10 culture and the section of UV metagenesis (5 s UV irradiation). SPV reactions were performed in accordance with the methods described in the section of optimizing the sample size for the SPV reaction using 15 µL samples without prior water removal (*n* = 3). Mutant lines that presented relative SPV reactivity values significantly higher than those of the naïve strain were subjected to fatty acid analysis by GC-MS (Yang et al. 2010) to confirm the DHA contents were significantly higher than those of the naïve strain. Statistical analysis was performed using a t-test with the level of significance set at *p* = 0.01.

## Results

Supplemental Figure S1 illustrates the correlation between UV exposure time and the mortality rate of BL10 cells under experimental conditions. A 5-second UV exposure yielded a mortality rate of 65%, which falls within the 60–80% range deemed optimal for UV mutagenesis (Pora and Zhou 2012). Extending the exposure time to beyond 10 s increased the mortality rate to 100%. Thus, 5-second UV exposure was selected for all subsequent UV mutagenesis experiments on BL10 cells.

**Fig. 1:**
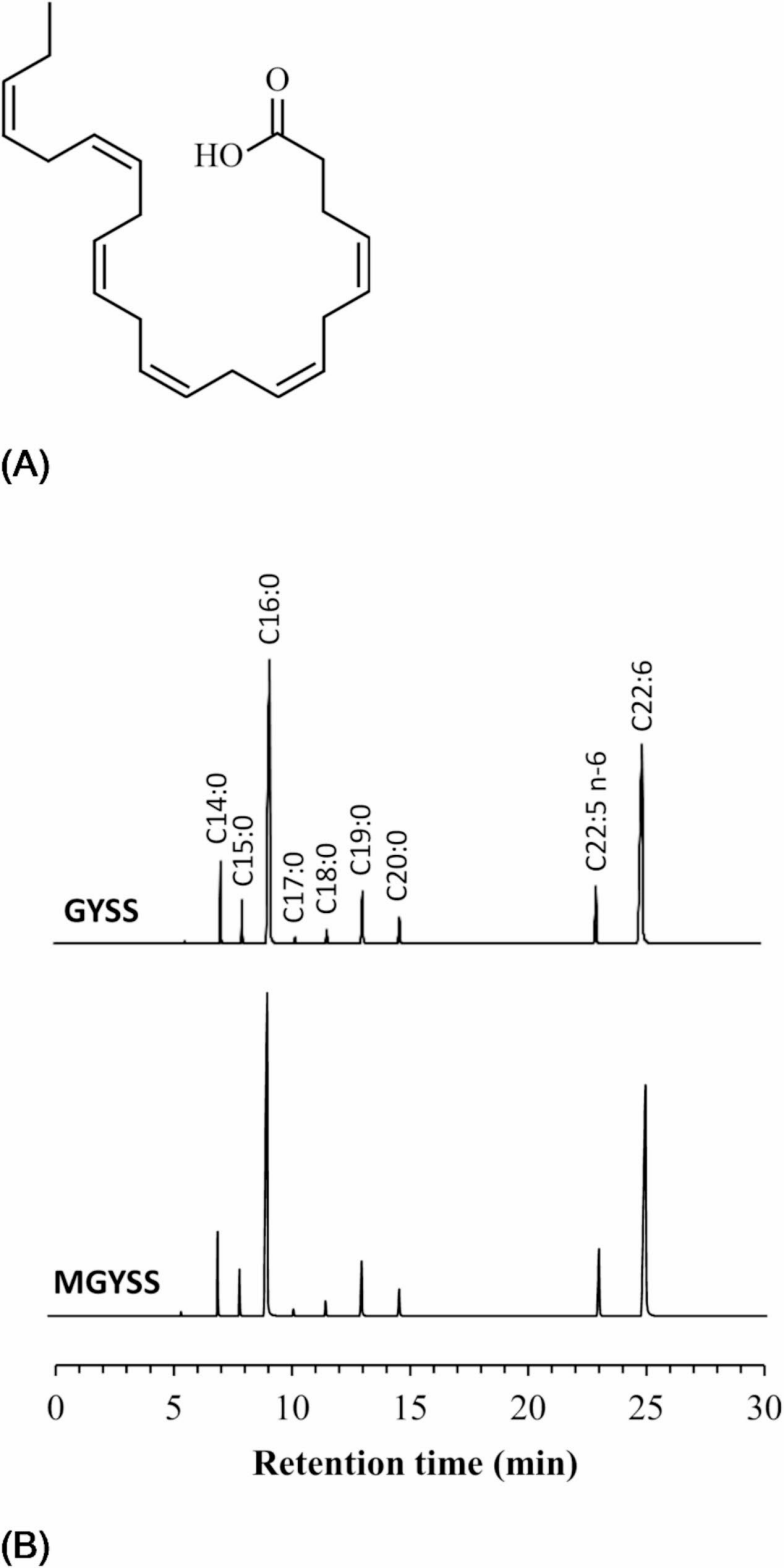
**A** Chemical structure of DHA; **B** Fatty acid profiles of BL10 cultivated in GYSS medium or modified GYSS (MGYSS) medium. Cultivation was performed in a 50 mL test tube containing 5 mL medium at 27 °C under orbital shaking at 150 rpm. Seven fatty acids were detected in the BL10, including C14:0, C15:0, C16:0, C17:0, C18:0, C22:5n − 6 (n-6 DPA) and C22:6 (DHA). C19:0 and C20 were incorporated in the BL10 as internal standards

The fatty acid profile of BL10 was remarkably simple, regardless of whether they were cultivated in GYSS or MGYSS medium. As shown in Fig. 1, the BL10 presented only two types of unsaturated fatty acid: DHA and n-6 DPA. The presence of C19:0 and C20:0 in GC-MS chromatograms can be attributed to internal standards, which were added at concentrations of 2% and 1% of the dry biomass, respectively.

Table 1(A) lists the weight percentages of DHA and n-6 DPA based on the signal intensities of DHA and n-6 DPA relative to those of C19:0 and C20:0, while accounting for the biomass from GYSS cultivation (15.14 gL^− 1^) and MGYSS cultivation (15.08 gL^− 1^). BL10 cultured in MGYSS medium (using only sea salt) yielded more DHA and n-6 DPA than did the BL10 cultured in GYSS (replacing 90% of the sea salt with sodium sulfate). Table 1(A) also lists the absolute concentrations of C–C double bonds attributable to the two unsaturated fatty acids, based on measured DHA and DPA concentrations.

If we assume that DHA and n-6 DPA are the main sources of C = C double bonds in BL10, and further assume that SPV reactivity is a faithful reflection of differences in C–C double bond content, then the SPV reactivity of MGYSS-BL10 cultured should exceed that of GYSS-BL10. Moreover, the relative SPV reactivity of MGYSS-BL10 vs. GYSS-BL10 should closely match the relative number of C–C double bonds in the culture samples. Table 1(B) lists relative SPV reactivity values as a function of sample volume (10, 15, 20, and 25 µL) with or without the removal of extracellular water prior to the SPV reaction. As expected, the relative SPV reactivity values exceeded 100% in all groups, regardless of reaction conditions. This means that the SPV reactivity of MGYSS-BL10 exceeded that of GYSS-BL10. This also means that accurate measurements of DHA production can be obtained without the need to perform centrifugation for the removal of extracellular water.

**Table 1:** Comparative analysis of *Aurantiochytrium limacinum* BL10 cultures in different media and sample preparation conditions

(A)		
	MGYSS	GYSS
[DHA](gL^− 1^)	2.857 ± 0.043	2.550 ± 0.277
[DPA] (gL^− 1^)	0.499 ± 0.009	0.402 ± 0.033
[C = C] (M)	0.060 ± 0.001	0.053 ± 0.006
RCC*	111.1 ± 7.4%	

(B)		
Vol	RSR** no water rem.	RSR*** water rem.
25 µL	105.0 ± 0.5%	105.7 ± 3.9%
20 µL	108.1 ± 1.9%	101.9 ± 2.1%
15 µL	112.5 ± 3.2%	104.1 ± 2.3%
10 µL	101.0 ± 2.8%	113.5 ± 2.3%

*Relative C–C double bond concentration

**Relative SPV reactivity without water removal

***Relative SPV reactivity with water removal

(A) Concentrations of DHA and n-6 DPA and the number of C–C double bonds attributable to DHA and n-6 DPA in BL10 cultivated in GYSS or modified GYSS (MGYSS) medium. Relative C–C double bond concentrations (MGYSS/ GYSS) are also listed. (B) SPV reactivity of MGYSS culture relative to GYSS culture under various sample volumes (10, 15, 20, or 25 µL) with or without extracellular water removal prior to the SPV reaction.

The relative SPV reactivity values of 15 µL samples without water removal most closely matched the relative number of C–C double bonds in Table 1(A). Thus, we adopted this method for all subsequent SPV reactions used for the screening of UV-mutant BL10 strains.

**Fig. 2:**
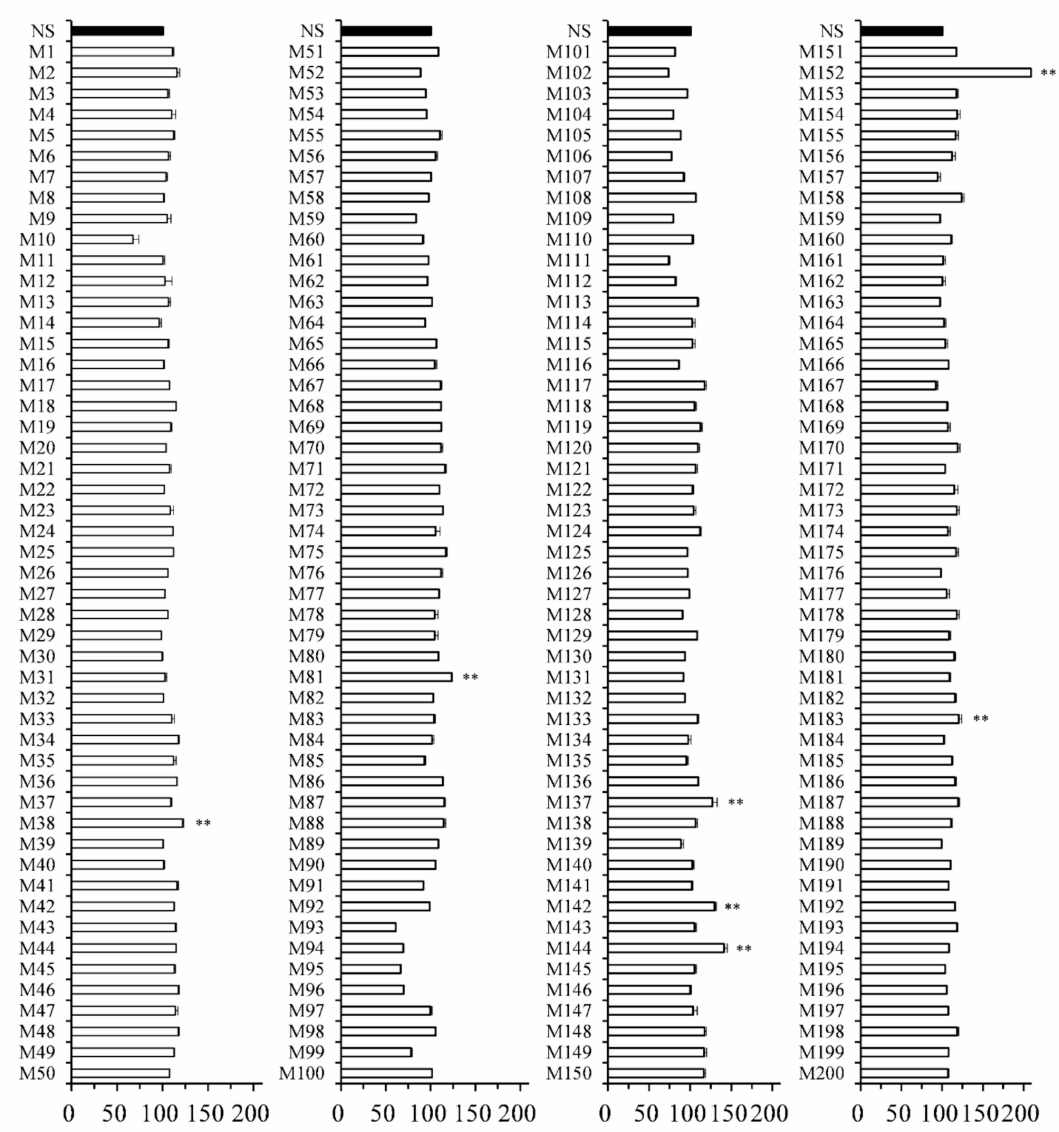
Results of SPV reaction analysis of BL10 lines generated via UV mutagenesis. Cultivation was performed in a 50 mL test tube containing 5 mL GYSS medium at 27 °C under orbital shaking (150 rpm) for three days. Following cultivation, the supernatant was removed via centrifugation (5000 x g, 5 min) to be replaced with deionized water to the original volume for resuspension of the pellet. SPV reactions were performed on 15 µL samples (*n* = 3 replicates)

**Fig. 3:**
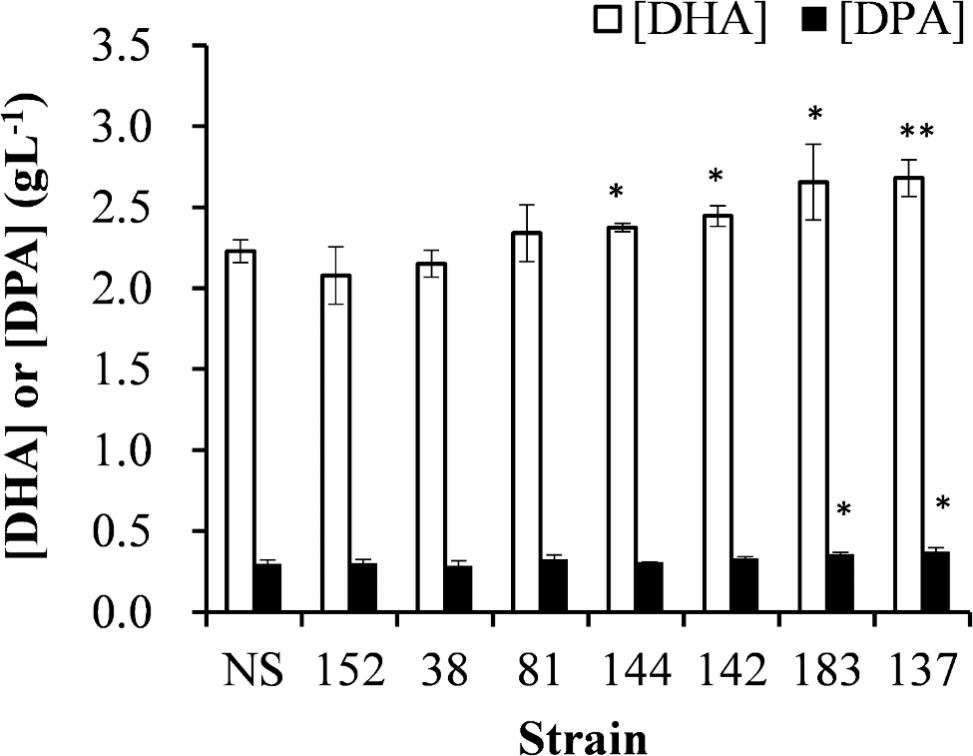
DHA and n-6 DPA content in 7 mutant lines with high SPV reactivity. Cultivation was performed in a 50 mL test tube containing 5 mL GYSS medium at 27 °C under orbital shaking (150 rpm) for three days. Following cultivation, the supernatant was removed via centrifugation (5000 x g, 5 min), followed by washing with deionized water and freeze-drying. The dry biomass weight was measured, and C19:0 and C20:0 as internal standards, which were added at concentrations of 2% and 1% of the dry biomass. Following the completion of saponification and esterification reactions, fatty acid composition analysis was performed using GC-MS. NS refers to the naïve strain

As shown in Fig. 2, the 200 strains (M1 ~ M200) were divided into four batches of 50 strains each prior to SPV reactivity analysis. Overall, we identified 7 strains with SPV reactivity significantly exceeding that of the naïve strain (NS), including M38, M81, M137, M142, M144, M152, and M183 (*P* < 0.01). As shown in Fig. 3, subsequent GC-MS analysis of those 7 strains revealed four strains with significantly elevated DHA concentrations (M137, M142, M144, and M183) and 2 strains with significantly elevated n-6 DPA concentrations (M137 and M183).

**Fig. 4:**
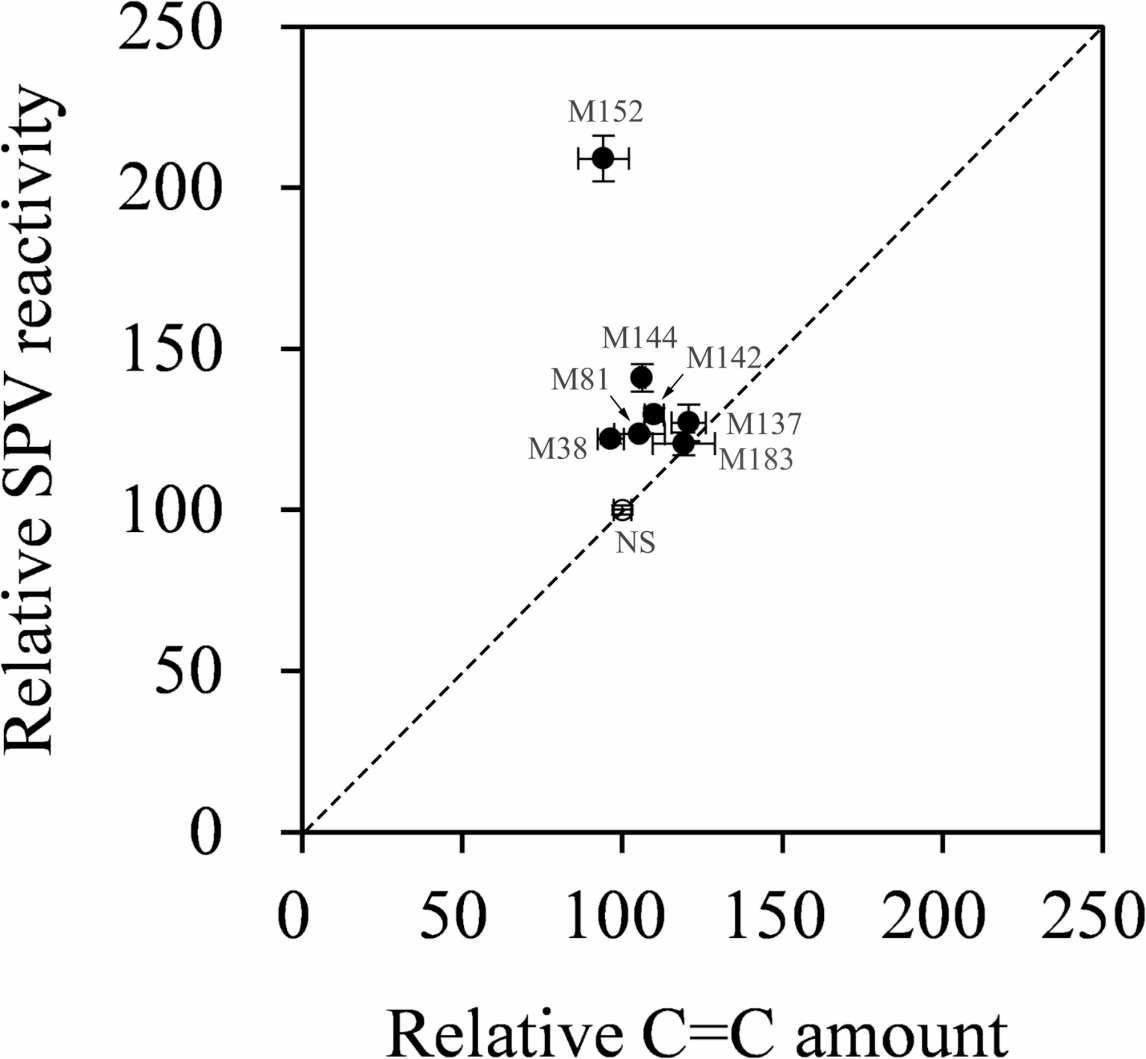
Cross-comparisons of the relative number of C–C double bonds and relative SPV reactivity. Normalization was conducted in 7 mutant lines with high SPV reactivity using the number of C–C double bonds and relative SPV reactivity of the naïve strain (NS)

Comparisons were also performed on relative SPV reactivity (normalized to 100% using the naïve strain) as well as the relative number of C–C double bonds from DHA and n-6 DPA (normalized to 100% using the naïve strain). As shown in Fig. 4, the relative SPV reactivity of 5 of the 7 strains significantly higher than the relative number of C–C double bonds, indicating that SPV reactivity tended to overestimate the DHA/DPA content in UV-mutant lines of *Aurantiochytrium limacinum*.

## Discussion

A high-throughput SPV-based screening system for the identification of *Aurantiochytrium* strains with high DHA content could have important commercial implications. Establishing a reliable screening system of high efficiency would require the optimization of SPV reaction conditions (e.g., sample volume). The system developed in the current study identified 7 mutant strains presenting high SPV reactivity. Subsequent GC-MS analysis confirmed that four of those strains yield large quantities of DHA.

Despite its effectiveness in high-throughput analysis, our SPV-based screening system has several inherent limitations. The specificity of the SPV-reaction is constrained by its reliance on the detection of C=C double bonds, which prevents clear differentiation between DHA and other polyunsaturated fatty acids. This lack of specificity increases the risk of false positives in the detection of DHA, particularly when dealing with strains that accumulate other polyunsaturated fatty acids.

Detection performance can also be affected by the presence of interfering compounds containing C=C double bonds (e.g., squalene and carotenoids), which occur naturally in *Aurantiochytrium*. Squalene is a colorless triterpene containing multiple C=C double bonds, which could impact SPV measurements. Carotenoids, which protect against oxidative stress, contain numerous conjugated double bonds capable of reacting with the SPV reagent. These interference effects can be mitigated by employing appropriate sample dilutions, including proper controls, and validating results using complementary analytic methods, such as GC-MS.

The integrity of fatty acid compounds is highly dependent on proper sample preparation. Critical steps include managing the reaction temperature to prevent thermal degradation, minimizing light exposure to avoid photo-oxidation, and limiting exposure to air to reduce oxidative damage. Consistent testing conditions are also essential, including standardized sample volumes, cell incubation density, centrifugation conditions, and the growth phase during which cells are harvested. These considerations are particularly important when dealing with *Aurantiochytrium* samples, as their high DHA content makes them particularly susceptible to degradation during preparation and storage.

The optimal sample volume is determined by the nature of the sample, the sensitivity of the detection method, and the analysis objectives. In the current study, sample volume was optimized by comparing the relative concentration of C–C double bonds and relative SPV reactivity at various volumes (10, 15, 20, and 25 µL). The effects of water removal on the SPV reaction varied with sample volume. Samples with water removal presented a vague negative correlation between relative SPV reactivity and volume with a slight upturn at 25 µL. Samples without water removal presented a parabolic relationship, with the optimal point at 15 µL, indicating the point at which the relative SPV reactivity (112.5 ± 3.2%) most closely matched the target value (111.1 ± 7.4%). Deviations from this volume increased the discrepancy between the two measured values. Thus, 15-µL samples without water removal were selected for subsequent fatty acids analysis in BL10 cultures. A previous research (Anschau et al. 2017) used 20-µL samples in the analysis of total lipids in lyophilized oleaginous microorganisms. (McMahon et al. 2013) used 100-µL samples in the analysis of total lipids in human meibomian gland secretions (meibum).

Water removal is an important step in optimizing SPV-based methods. A small quantity of water is necessary for lipid ester hydrolysis, while excessive water content can dilute the reaction and thereby impair detection performance. A low water-to-acid ratio is crucial for optimal color development and accurate lipid quantification. Higher water-to-acid ratios can impair color development through dilution effects and competition for reactions with sulfuric acid. (Anschau et al. 2017) reported minimal color development with a water-to-acid ratio of 1:3, while higher water-to-acid ratios (1:1 and 3:1) resulted in no significant color development at the target wavelength.

Interestingly, water removal did not have a significant influence on DHA detection in the experiments on BL10 cultures. This result can be attributed to the fact that most of the sample volume consisted of water from the culture medium, which likely provided an adequate amount of water for lipid ester hydrolysis without requiring additional adjustments, making water removal unnecessary.

By optimizing the sample volume and retaining the culture’s natural water content, we developed a more streamlined and efficient SPV-based screening method for identifying *Aurantiochytrium* strains with high DHA content.

From a total of 200 mutant cell lines, our high-throughput screening system enabled the rapid identification of 7 strains exhibiting elevated SPV reactivity. Due to limitations on the availability of algal cultivation equipment in our laboratory, we had to divide the 200 mutant strains into 4 batches for SPV analysis. Nonetheless, the rapid SPV analysis of each batch was completed within one hour, and subsequent GC-MS analysis was completed within one working day. GC-MS analysis verified that four of the identified strains had elevated DHA levels and two strains had elevated n-6 DPA levels, compared to the naïve strain. Note however that the remarkable simple fatty acid profile of BL10 no doubt contributed to the ease with which these strains were identified.

One intriguing discovery in this study was the correlation between the relative SPV reactivity (normalized to 100% using the naïve strain) and the relative number of C–C double bonds corresponding to DHA and n-6 DPA (also normalized to 100%). Note that this comparison was meant to assess the reliability of the SPV method. We determined that the SPV reactivity method tended to overestimate the content of DHA/n-6 DPA in the mutant lines of *Aurantiochytrium limacinum*, based on the fact that SPV reactivity significantly exceeded the number of C–C double bonds in 5 of the 7 identified strains. This may indicate the presence of other unsaturated fatty acids. Previous studies reported on the presence of other unsaturated fatty acids in *Aurantiochytrium* sp., including eicosapentaenoic acid (EPA), oleic acid (Song et al. 2022) and palmitoleic acid (Heggeset et al. 2019), albeit in relatively low quantities.

It is important to consider the possible effects of environmental variables (e.g., UV light exposure) on the quantity of unsaturated fatty acids. UV exposure was shown to increase EPA and DHA content by more than 30% in *Pavlova lutheri* (Blasio and Balzano 2021). UV exposure was also shown to increase oleic acid content in *Thalassiosira weissflogii* (Durif et al. 2015) and palmitoleic acid content in the marine green microalgae, *Platymonas subcordiformis* (Huang et al. 2020).

It is possible that the inflated estimates of DHA/n-6 DPA content in mutant lines was due to an increase in the quantity of other fatty acids (e.g., EPA, oleic acid, palmitoleic acid). Note that GCMS analysis imposes a minimum detection limit, which means that any unsaturated fatty acids falling below this threshold would go undetected. Our GCMS results detected no fatty acids other than DHA and n-6 DPA in mutant lines (data not shown). Even if other unsaturated fatty acids were present in the samples, we can safely assume that they existed at concentrations below the detection limit, which is too low to have a detectable effect on the results. Therefore, we can dismiss this possibility.

The inflated estimates of DHA/n-6 DPA content after UV treatment could perhaps be explained by the presence of carotenoids, which are abundant in C–C double bonds. *Aurantiochytrium* sp. is a microorganism known to produce carotenoids as a form of protection against damage due to light or oxidative stress (Reis-Mansur et al. 2019). In many desert plants, carotenoid levels increase under UV stress (Salama et al. 2011). One previous study on *Aurantiochytrium* sp. TZ209 reported that high light intensity promoted the synthesis of carotenoids, which were likely contributors to changes in the pigmentation of this microalgae (Yin et al. 2023). Those findings suggest that elevated carotenoid levels might manifest as a change in the color of *Aurantiochytrium* sp; however, none of the mutant lines with elevated SPV reactivity presented observable color variations, thereby ruling out this possibility.

It appears that the interfering molecules are not unsaturated fatty acids or carotenoids; however, they may well be compounds induced by UV radiation. Squalene is a colorless triterpene that serves as a precursor in the production of carotenoids and steroids. Researchers have previously identified *Aurantiochytrium* sp. as a candidate vehicle for squalene production (Nakazawa et al. 2012; Patel et al. 2019b; Zhang et al. 2019). Squalene contains multiple C–C double bonds (Micera et al. 2020), which can be identified via SPV analysis. Squalene also acts as a natural antioxidant protecting *Aurantiochytrium* sp. cells from reactive oxygen species (Patel et al. 2019b). Since UV radiation can induce oxidative stress, it is plausible that UV exposure could stimulate squalene production as a protective mechanism.

Despite these limitations, our findings indicate that SPV analysis is a valid high-throughput screening method for the identification of *Aurantiochytrium* strains with high DHA content. In terms of time, effort, and cost effectiveness, SPV analysis is far more efficient than conventional screening methods (e.g., gas chromatography and fluorescent staining methods) by allowing the rapid screening of multiple mutant strains simultaneously.

The SPV-based screening method offers distinct advantages over other analytic techniques in terms of time, effort, and cost efficiency. Although gas chromatography remains the gold standard for fatty acid analysis, it is ill-suited to high-throughput screening, due to the need for extensive sample preparation, extended analysis times, and high operational costs due to specialized equipment and consumables.

The proposed screening system is ideally suited to large-scale screening applications, as demonstrated by the completion of 50-strain batches within one hour. Using the GC-MS method for the analysis of 200 samples would require 27 days. Combining SPV analysis with GC/MS validation reduced this to only 4 days, representing an 85% reduction in processing time. This level of efficiency can be attributed to minimal sample preparation, rapid high-throughput screening owing to rapid reaction kinetics, straightforward data interpretation, and the capacity to analyze multiple samples simultaneously.

Lipophilic fluorescent staining (e.g., Nile Red and BODIPY) has been widely used for the analysis of neutral lipids in green algae, such as *Chlorella ellipsoidea* and *Chlorococcum infusionum* (Satpati and Pal 2015) as well as *Nannochloropsis oceanica* (Südfeld et al. 2021). However, fluorescent staining cannot differentiate between saturated and unsaturated fatty acids, and they provide limited specificity when applied to polyunsaturated fatty acids, such as DHA.

The SPV method provides superior precision by specificity by detecting the C = C double bonds characteristic of unsaturated fatty acids. It also simplifies sample preparation and data interpretation, while eliminating the need for specialized fluorescence detection equipment. Fluorescence microscopes and flow cytometers can cost tens to hundreds of thousands of dollars and fluorescent dyes, such as Nile Red ($240 - $8600 per gram). By contrast, the SPV method utilizes relatively inexpensive spectrophotometers and affordable reagents. Specificity, simplicity, cost-effectiveness and high-throughput capability makes the SPV method particularly suitable for screening *Aurantiochytrium* strains for DHA content in commercial applications.

The successful implementation of SPV requires precise control over growth conditions, including reaction temperature and exposure to light and air. It also requires consistent testing conditions, such as sample volume, cell incubation density, centrifugation conditions, the growth phase during which cells are harvested, and timely sample processing.

The effectiveness of this method can be further enhanced through integration within a multi-tiered analytical system, where SPV screening serves as an initial high-throughput filter, followed by GC-MS for the verification of promising strains. This multi-step approach mitigates the shortcomings of each method while leveraging their respective strengths: SPV provides rapid screening but lacks compound specificity, whereas GC-MS provides detailed fatty acid profiles but requires extensive sample preparation.

Combining optimized operating conditions with the strategic integration of complementary analytic methods balances speed with precision. This robust framework is particularly well-suited to commercial applications requiring large-scale screening.

In summary, this study demonstrates the potential of SPV analysis for the high-throughput screening of *Aurantiochytrium sp.* to identify novel candidate strains with high-DHA content. The advances are crucial to the sustained production of DHA-rich algal oils at commercial scales.

## Electronic supplementary material

Below is the link to the electronic supplementary material.

Supplementary Material 1: Fig. S1. The measured lethality rate of BL10 under different UV irradiation times. Irradiation with the UV 254 light source of a laboratory UV colloidal transilluminator gel documentation system, the irradiation time is 0, 1, 10, 20, 30, 40, 50, 60, 70, 80, 90 and 100 s.

## Data Availability

The data and materials that support the findings of this study are available from the corresponding author, upon reasonable request.
