# Supplementary material for: Sulfo-phospho-vanillin method for screening Aurantiochytrium strains with high docosahexaenoic acid levels
Source: AMB Express. 2025 Mar 20;15:52. doi: 10.1186/s13568-025-01859-9 (PMC11925841; doi:10.1186/s13568-025-01859-9)
Supplement: Supplementary file 1 — Supplementary Material 1: Fig. S1. The measured lethality rate of BL10 under different UV irradiation times. Irradiation with the UV 254 light source of a laboratory UV colloidal transilluminator gel documentation system, the irradiation time is 0, 1, 10, 20, 30, 40, 50, 60, 70, 80, 90 and 100 s. [file 13568_2025_1859_MOESM1_ESM.docx]

**Supplemental figure S1**
